# WHO/ICC Classification for Myelodysplastic Neoplasms/Syndromes Performs Better for Subtype Cytomorphological Diagnosis?

**DOI:** 10.3390/diagnostics14151631

**Published:** 2024-07-29

**Authors:** Ana Isabel Vicente, Irene Luna, Juan Carlos Ruiz, María José Remigia, Andrés Jerez, Rafael Lluch, Inmaculada Llopis, María Josefa Marco, Carmen Benet, Carmen Alonso, María Dolores Linares, Luis Serrano, María Teresa Orero, Francisco José Ortuño, María Leonor Senent

**Affiliations:** 1 Hospital Universitario y Politécnico La Fe, 46026 Valencia, Spain; senent_leo@gva.es; 2 Instituto de Investigación Sanitaria La Fe, 46026 Valencia, Spain; 3 Departamento de Metodología de las Ciencias del Comportamiento, Facultad de Psicología, Universidad de Valencia, 46010 Valencia, Spain; juan.c.ruiz@uv.es; 4 Servicio de Hematología, Hospital Clínico Universitario, 46010 Valencia, Spain; remigiamj@gmail.com; 5 Servicio de Hematología, Hospital General Universitario Morales Meseguer, 30008 Murcia, Spain; anjecayu@gmail.com (A.J.); fortunog@sehh.es (F.J.O.); 6 Servicio de Hematología, Hospital Universitario de la Ribera, 46600 Valencia, Spain; lluch_rafgar@gva.es (R.L.); llopis_inm@gva.es (I.L.); 7 Servicio de Hematología, Hospital Universitario Dr. Peset, 46017 Valencia, Spain; jmarcobuades@gmail.com; 8 Servicio de Hematología, Hospital Arnau de Vilanova, 46015 Valencia, Spain; benet_car@gva.es (C.B.); alonso_carpri@gva.es (C.A.); 9 Servicio de Hematología, Hospital General Universitario de Castellón, 12004 Castelló de la Plana, Spain; lolalinares@hotmail.com (M.D.L.); serrano.picazo.luis@gmail.com (L.S.); 10 Servicio de Hematología, Consorcio Hospital General Universitario, 46014 Valencia, Spain; orero_may@gva.es; 11 CIBERONC, Instituto de Salud Carlos III, 28029 Madrid, Spain

**Keywords:** myelodysplastic syndromes, morphological evaluation, blast count concordance, WHO classification, ICC classification

## Abstract

The International Consensus Classification of Myeloid Neoplasms and Acute Leukemias (ICC) and the 5th edition of the WHO classification (WHO 2022) have refined the diagnosis of myelodysplastic syndromes (MDS). Both classifications segregate MDS subtypes based on molecular or cytogenetic findings but rely on the subjective assessment of blast cell percentage and dysplasia in hematopoietic cell lineages. This study aimed to evaluate interobserver concordance among 13 cytomorphologists from eight hospitals in assessing blast percentages and dysplastic features in 44 MDS patients. The study found fair interobserver agreement for the PB blast percentage and moderate agreement for the BM blast percentage, with the best concordance in cases with <5% BM blasts and >10% BM blasts. Monocyte count agreement was fair, and dysplasia assessment showed moderate concordance for megakaryocytic lineage but lower concordance for erythroid and granulocytic lineages. Overall, interobserver concordance for MDS subtypes was moderate across all classifications, with slightly better results for WHO 2022. These findings highlight the ongoing need for morphological evaluation in MDS diagnosis despite advances in genetic and molecular techniques. The study supports the blast percentage ranges established by the ICC but suggests refining BM blast cutoffs. Given the moderate interobserver concordance, a unified classification approach for MDS is recommended.

## 1. Introduction

To further refine and standardize the diagnosis of myelodysplastic syndromes (MDS), two different proposals have recently been published: the International Consensus Classification of Myeloid Neoplasms and Acute Leukemias (ICC) [1] and the 5th version of the WHO classification (WHO 2022) [2]. Both classifications segregate the same three entities by molecular or cytogenetic findings, with similar biological and clinical features. 

However, the percentage of blast cells in bone marrow (BM) and peripheral blood (PB), and to a lesser extent, the number of dysplastic hematopoietic cell lineages are essential for defining the subtypes of MDS in both classifications but prone to a great degree of subjectivity. Further, several relevant discordances between ICC and WHO classification systems with potential clinical consequences in patient management depend on these two morphological variables. 

The main aim of this study was to evaluate, in a real-world setting, the degree of concordance among 13 cytomorphologists for assessing the percentage of blasts and dysplastic features in 44 patients with MDS and determine their impact on the performance of the WHO 2022 and ICC classifications. 

## 2. Materials and Methods

### 2.1. Patients and Samples

Thirteen hematologists from eight hospitals in the Comunidad Valenciana, Spain, participated in the study. Each center was a departmental hospital, covering the healthcare of 200,000 to 380,000 people. Seven of them were also University hospitals, five were tertiary-care referral centers with units specialized in hematological cytomorphologic diagnosis, and only one was an MDS Foundation Center of Excellence. Each participating center contributed PB and BM smears from six patients clearly diagnosed with MDS according to WHO 2016 criteria. Four cases were excluded due to poor quality staining (three) and extreme hypocellularity (one). Thus, PB and BM smears obtained at diagnosis from 44 individuals were finally considered evaluable and blindly reviewed by all observers. Patients were classified according to the WHO 2016 [3], WHO 2022, and ICC classifications based only on the morphological data. Cytogenetic and molecular data were not considered for the purposes of the study. 

### 2.2. Morphological Studies

At least four smears from each patient included in the study were available for blind and independent microscopic review. One PB and two BM May–Grünwald–Giemsa (MGG)-stained smears were used along with one Prussian blue-stained BM smear. The variables analyzed were percentages of blasts and monocytes, the presence of dysplasia in each hematopoietic lineage, the percentage of ring sideroblasts, and the proposed diagnostic classification. 

The WHO 2016 recommendations for evaluating the morphological diagnosis of MDS were followed (Table 1). 

The WHO 2022 and ICC recommendations were subsequently centrally implemented (Table 2).

Blasts and ring sideroblasts were defined according to the consensus proposals of the International Working Group on Morphology of Myelodysplastic Syndromes (IWGM-MDS) [4].

In the WHO 2016 classification, the presence of 1% PB blasts recorded on at least two separate occasions with less than 5% BM blasts is diagnostic of unclassifiable MDS, whereas this implies MDS with excess blasts in the ICC. This does not affect the WHO 2022 classification. Cases with a single blast count of 1% were not considered to qualify for a distinct category since the observers did not have a second PB determination. PB and BM differential counts were performed on at least 200 and 500 cells, respectively. To assess dysplasia, at least 200 neutrophils, 200 erythroid precursors, and 30 megakaryocytes were evaluated in bone marrow.

Information on the hemoglobin level and white blood cell and platelet counts was available for observers when performing the morphological review, whereas clinical and genomic data were not provided to them. 

All variables were recorded in specifically designed forms. No attempt to reach a consensus agreement on cases with discrepant results was made.

### 2.3. Statistical Analysis

The quantitative variables assessed were the percentages of monocytes and blasts in PB, percentages of blasts in BM, and percentages of dysplastic cells of erythroid, granulocytic, and megakaryocytic lineages in PB and/or BM. Quantitative variables were evaluated as continuous as well as after their categorization. The qualitative variables analyzed were the presence or absence of dysplasia greater than 10% in any hematopoietic cell lineage and the subtype of MDS classification according to the three classifications. 

The degree of correlation between observers of the quantitative variables was evaluated using the intraclass correlation coefficient (ICCo) [5]. The ICCo has two advantages over Spearman’s correlation coefficient: it adjusts for the effects of the scale of measurements and also allows the assessment of agreement between more than two observers. The ICCo must be interpreted as follows: <0.30 indicates poor agreement, 0.31–0.50 indicates fair agreement, 0.51–0.70 indicates moderate agreement, 0.71–0.90 indicates good agreement, and >0.90 indicates very good agreement. 

The generalized kappa statistic for multiple raters (κ) was used to evaluate the degree of concordance between observers in qualitative and categorized quantitative variables. The generalized κ statistic should be interpreted as follows: <0.20 indicates poor agreement, 0.21–0.40 indicates fair agreement, 0.41–0.60 indicates moderate agreement, 0.61–0.80 indicates strong agreement, and >0.80 indicates almost perfect agreement. 

All analyses were performed using the statistical package IBM SPSS, version 28.0 (IBM Corporation, Armonk, NY, USA).

## 3. Results

The degree of concordance between observers for the different morphological characteristics evaluated is depicted in Table 3. 

The level of agreement regarding the morphological subtype according to the WHO 2016, WHO 2022, and ICC criteria is shown in Table 4.

### 3.1. Interobserver Concordance Regarding Blast Cell Count 

Interobserver blast cell percentage agreement in PB considered as a continuous variable was fair (ICCo 0.45). Stratifying into three (<2%, 2–4%, 5–9%) categories, the worst result was for the 2–4% range and the best concordance was seen in the <2% blast group. 

There was moderate but statistically significant agreement in the percentage of blast cells in BM considered as a continuous variable (ICCo 0.68). When considering this variable by categories, the best result is for the <5% group, and the worst, again, for the intermediate group (5–9%).

### 3.2. Interobserver Concordance Regarding Monocyte Cell Count in Peripheral Blood

There was fair agreement in the percentage of monocyte count in PB considered as a continuous variable (ICCo 0.47) as well as stratifying the variable into the two categories (<10% and ≥10%) used in the WHO classification.

### 3.3. Interobserver Concordance Regarding the Assessment of Dysplasia

When the presence of dysplasia of the three hematopoietic cell lines was studied as a continuous variable, the concordance between observers was moderate for the megakaryocytic lineage and low for the erythroid and granulocytic lineages in all instances (ICCo 0.63, ICCo 0.44, and ICCo 0.45, respectively). When those variables were stratified according to the 10% cutoff point required by the WHO and ICC criteria, the agreement was fair for the megakaryocytic and erythroid lineages but poor for the granulocytic lineage.

### 3.4. Reproducibility of WHO 2016, WHO 2022, and ICC-Defined Subtypes of Myelodysplastic Syndromes

The overall interobserver concordance was moderate for the three classifications (κ 0.49 for WHO 2016, κ 0.54 for WHO 2022, and κ 0.48 for ICC). As expected, given the poor agreement in the BM blast count group between 5 and 9%, the worst result was observed in the SMD-EB (ICC)/SMD-IB1 (WHO 22) group.

## 4. Discussion

A better understanding of the pathophysiology of hematological diseases due to advances in genetic techniques is allowing for more accurate subclassification of myeloid neoplasms. Within the MDS, three entities can be genetically defined; however, the blast cell percentage is essential to subclassifying the different subtypes of MDS, even those with defined cytogenetic alterations. Moreover, morphological recognition of blasts and dysplasia is still needed [6] to separate entities such as clonal cytopenia of undetermined significance (CCUS) from MDS or for the differential diagnosis with paroxysmal nocturnal hemoglobinuria (PNH), aplastic anemia, and other non-neoplastic clinical pictures.

To address both dysplasia evaluation and blast identification, the International Working Group on Morphology of myelodysplastic syndrome has elaborated consensus proposals for the definition of myeloblasts, promyelocytes, monoblasts, promonocytes, and ring sideroblasts [4,7] and recommendations for interpretation of atypical features of megakaryopoiesis [8], granulopoiesis [9], and erythropoiesis [10]. However, cytomorphology remains subjective. 

The current study was designed to assess interobserver variability among a heterogeneous group of cytomorphologists in the recognition of blast cells and dysplastic features and their influence on the diagnosis of MDS according to the classification criteria of the WHO 2016, WHO 2022, and ICC. 

When addressing the PB blast count as a continuous variable, fair agreement was found, although reproducibility was poor when categorizing between 2–4% and 5–9%. For this reason, we believe that the categorization proposed by the ICC would be more appropriate to homogenize the subclassification of MDS. On the other hand, there was better concordance in the range of <2% of blasts. According to the ICC, the count of 1% of blasts in two consecutive determinations is a diagnosis of MDS-EB. It is difficult to check whether this is an appropriate decision as this involves a very small number of cases. 

The agreement in BM percentage blast cells was better than that found in blood, especially in cases with less than 5% blasts and those with >10% blasts. Therefore, the best concordance in the diagnosis is reached in cases with low blasts and in the subtypes with increasing blasts type 2 of the WHO 2022 and in the MDS/AML of the ICC. The groups with increased myeloblasts are associated with worse outcomes, but the exact cutoff is not clear [11]. 

The cutoff for absolute monocytosis has been lowered from 1.0 × 10^9^/L to 0.5 × 10^9^/L in both classifications for CMML diagnosis. Our agreement on the monocyte count was fair; therefore, we believe that the requirement of a molecular or immunophenotypic alteration is a reasonable diagnostic support.

Addressing dysplasia, we found the best reproducibility in the megakaryocytic lineage. Dyserythropoiesis and dysgranulopoiesis achieved only fair agreement. Regarding the granulocytic series, differences between MGG stain methods might imply this variability. Although ringed sideroblasts showed very good reproducibility, they have lost significance in favor of SF3B1 mutations. Finally, differing from other groups [12,13], our study showed good concordance for MDS-SLD and moderate concordance for MDS-MLD. However, the WHO 2022 classification dispenses with this specification.

When considering the different MDS classifications, moderate agreement was obtained in each of them, yet slightly better concordance for WHO 2022 was observed. Giagounidis and Haase [14] reported high concordance in making a diagnosis of MDS, but that review was undertaken by highly experienced experts. In our study, closer to real-life diagnosis, we observed only moderate agreement. 

Genetic and molecular findings can modify the diagnosis, prognosis, and even treatment of MDS. In this regard, Zhang et al. [15] reported significantly shorter median survival for MDS with biallelic *TP53* inactivation and MDS with fibrosis compared with other MDS subtypes. Recently, Huber et al. proposed replacing the MDS classification with a more genetically based approach [16]. However, even in specialized units, genetic and molecular results usually have a turnaround time of weeks, therefore leaving urgent or initial decisions to be made on morphologic grounds. 

In conclusion, with the current approach to the diagnosis of MDS, morphological evaluation of blast cells and recognition of dysplasia remain essential. The results of this study support the ranges established by the ICC in the evaluation of blasts in peripheral blood, but the cutoff point of blasts needs to be refined, especially in the bone marrow. 

Considering the interobserver concordance demonstrated in our study, both classifications can be applied with only moderate confidence, although from our point of view, an effort to return to a single classification is mandatory.

## Figures and Tables

**Table 1:** WHO 2016 morphological classification.

	Dysplastic Lineages	Cytopenias	Ring Sideroblasts (%)	BM/PB Blasts
**MDS with single lineage dysplasia**	1	1–2	<15%	<5% BM <1% PB No Auer rods
**MDS with multilineage dysplasia**	2–3	1–3	<15%	<5% BM <1% PB No Auer rods
**MDS with ring sideroblasts**			≥15%	<5% BM <1% PB No Auer rods
MDS-RS-SLD	1	1–2		
MDS-RS-MLD	2–3	1–3		
**MDS with excess blasts** MDS-EB-1	1–3	1–3	None or any	5–9% BM 2–4% PB No Auer rods
MDS-EB-2				10–19% BM 5–19% PB or Auer rods
**MDS-unclassifiable** With SLD and pancytopenia	1	3	None or any	BM < 5% PB < 1% No Auer rods
With 1% PB blasts	1–3	1–3	None or any	BM < 5% PB = 1% No Auer rods

MDS, myelodysplastic syndrome; BM, bone marrow; PB, peripheral blood; RS, ring sideroblasts; SLD, single lineage dysplasia; MLD, multilineage dysplasia; EB, excess blasts.

**Table 2:** WHO 2022 and ICC morphological classifications.

Entity Name WHO 2022	BM/PB Blasts	Entity Name ICC	Dysplastic Lineages	Cytopenias	BM/PB Blasts
**MDS with low blasts**	<5% BM <2% PB	**MDS, NOS with single lineage dysplasia**	1	1–3	<5% BM <2% PB
**MDS with low blasts and ring sideroblasts**	<5% BM <2% PB ≥15% ring sideroblasts	**MDS, NOS with multilineage** **dysplasia**	2–3	1–3	<5% BM <2% PB
**MDS with increased blasts 1**	5–9% BM 2–4% PB	MDS with excess blasts *	1–3	1–3	5–9% BM 2–9% PB
**MDS with increased blasts 2**	10–19% BM 5–19% PB or Auer rods	**MDS/AML**	1–3	1–3	10–19% BM or PB

MDS, myelodysplastic syndrome; BM, bone marrow; PB, peripheral blood; NOS, not otherwise specified; AML, acute myeloid leukemia. * The presence of 1% PB blasts confirmed on two separate occasions also qualifies for MDS with excess blasts.

**Table 3:** Statistical analyses of interobserver degree of agreement regarding morphological features.

	κ (*p* Value)		κ (*p* Value)	ICCo
**Blasts in peripheral blood (%)**				
<2	0.36 (*p* < 0.001)			
2–4	0.19 (*p* < 0.001)			
5–9	0.29 (*p* < 0.001)			
≥10	NA			
Overall kappa	0.28 (*p* < 0.001)			
As a continuous variable				0.45
**Monocytes (%)**				
<10	0.32 (*p* < 0.001)			
≥10	0.32 (*p* < 0.001)			
Overall kappa	0.32 (*p* < 0.001)			
As a continuous variable				0.47
**Blasts in bone marrow (%)**				
<5	0.62 (*p* < 0.001)	<10	0.60 (*p* < 0.001)	
5–9	0.34 (*p* < 0.001)	≥10	0.60 (*p* < 0.001)	
10–19	0.58 (*p* < 0.001)			
Overall kappa	0.51 (*p* < 0.001)			
As a continuous variable				0.68
**Bone marrow granulocytic dysplasia**				
<10%	0.05 (*p* = 0.01)			
≥10%	0.05 (*p* = 0.01)			
Overall kappa	0.05 (*p* = 0.01)			
As a continuous variable				0.45
**Bone marrow megakaryocytic dysplasia**				
<10%	0.36 (*p* < 0.001)			
≥10%	0.36 (*p* < 0.001)			
Overall kappa	0.36 (*p* < 0.001)			
As a continuous variable				0.63
**Bone marrow erythroid**				
**dysplasia**				
<10%	0.34 (*p* < 0.001)			
≥10%	0.34 (*p* < 0.001)			
Overall kappa	0.34 (*p* < 0.001)			
As a continuous variable				0.44

ICCo, intraclass correlation coefficient; NA, not apply.

**Table 4:** Statistical analyses of interobserver degree of agreement regarding WHO 2016, WHO 2022, and ICC classifications.

Subtypes of Myelodysplastic Syndromes	κ (*p* Value)
**WHO 2016**	
MDS with single lineage dysplasia	0.68 (*p* < 0.001)
MDS with multilineage dysplasia	0.46 (*p* < 0.001)
MDS with ring sideroblasts SLD	0.01 (*p* = 0.63)
MDS with ring sideroblasts MLD	0.68 (*p* < 0.001)
MDS with excess blasts type 1	0.33 (*p* < 0.001)
MDS with excess blasts type 2	0.56 (*p* < 0.001)
Overall kappa	0.49 (*p* < 0.001)
**WHO 2022**	
MDS with low blasts	0.58 (*p* < 0.001)
MDS with low blasts and ring sideroblasts	0.77 (*p* < 0.001)
MDS with increased blasts 1	0.34 (*p* < 0.001)
MDS with increased blasts 2	0.56 (*p* < 0.001)
Overall kappa	0.54 (*p* < 0.001)
**ICC**	
MDS, NOS with single lineage dysplasia	0.55 (*p* < 0.001)
MDS, NOS with multilineage dysplasia	0.54 (*p* < 0.001)
MDS with excess blasts	0.35 (*p* < 0.001)
MDS/AML	0.57 (*p* < 0.001)
Overall kappa	0.48 (*p* < 0.001)

MDS, myelodysplastic syndrome; SLD, single lineage dysplasia; MLD, multilineage dysplasia; NOS, not otherwise specified; AML, acute myeloid leukemia.

## Data Availability

The raw data supporting the conclusions of this article will be made available by the authors on request.
